# Construct Diversity in Measures of Helicopter Parenting in Emerging Adulthood

**DOI:** 10.1177/21676968251407137

**Published:** 2026-01-09

**Authors:** Andrea L. Howard, Sydney Bota, Megan Lamb

**Affiliations:** 1 Department of Psychology, 6339 Carleton University, Ottawa, ON, Canada

**Keywords:** helicopter parenting, emerging adulthood, measurement, construct validity

## Abstract

Numerous instruments assessing helicopter parenting exist, but items within and across scales are diverse and often describe behaviors that are incongruent with basic construct criteria. We recruited *n* = 1,222 emerging adults to rate how well 54 item stems conformed to a broad provided description of helicopter parenting (i.e., definitional correspondence), and answer follow-up questions related to item frequency, personal experience with the behavior, and perceived parent motivations. Correspondence ratings varied widely. For 12 items, emerging adults who directly experienced a behavior had lower definitional correspondence ratings compared to those with no direct experience. Close to half of items were seen as helicopter parenting only if the behaviors were frequently-occurring. Definitional correspondence was highly correlated with perceived control and fear of failure motives, and negatively correlated with perceived support motives. We offer a provisional definition of helicopter parenting and offer suggestions for item selection and refinement in ongoing construct validation research.

Are parents stifling their children’s progress toward independence through excessive involvement during the transition to adulthood? The term *helicopter parenting* was coined in 1990 in a parenting handbook to describe a practice of hovering over and rescuing young children from trouble: “While today these ‘loving’ parents may feel they are easing their children’s path into adulthood, tomorrow the same children will be leaving home and wasting the first eighteen months of their adult life flunking out of college or meandering about” (Cline & Fay, 2006, pp. 21-22). In the years that followed, anecdotes about helicopter parenting flooded the media, often focused on college students whose parents struggled to let their kids navigate life on their own (e.g., Strauss, 2006).

Several research teams saw an urgent need to contribute empirical evidence to a conversation that appeared to be driving policy changes at schools and colleges (Bradley-Geist & Olson-Buchanan, 2014; LeMoyne & Buchanan, 2011; Padilla-Walker & Nelson, 2012; Schiffrin et al., 2014; Segrin et al., 2012). Each team designed multi-item scales based on descriptions of helicopter parenting in the press, the scholarly literature, and their own intuitions, administered those items to samples of undergraduates or their parents, and correlated scores from the resulting scales with other relevant constructs (e.g., other measures of parenting, academic achievement, and well-being). Dozens of subsequent studies used these measures, more scales were developed (e.g., Luebbe et al., 2018; Odenweller et al., 2014), including one short form (Jiao & Segrin, 2022), a consolidated measure (Schiffrin et al., 2019) and numerous applications outside of North America (e.g., Wang et al., 2021; Wu et al., 2023; Zhang et al., 2020). In the present study, we document the highly diverse and sometimes contradictory content appearing across and within scales, arising in part from a history of failing to establish the substantive validity of helicopter parenting—a coherent definition, scope of the construct, and item content—during scale development. Our goal is to explore the substantive validity of existing item content by soliciting emerging adults’ perspectives on how well items correspond with a broad description of helicopter parenting.

## Interim Definitions and Measurement of Helicopter Parenting

Broad measures of *parenting* in emerging adulthood (ages 18–25) share similarities with those in adolescence, notably emotional warmth and autonomy support (Grolnick et al., 1991), that continue to be beneficial in emerging adulthood (e.g., Budescu & Silverman, 2016; Nelson et al., 2015). However, there is disagreement and concern over parent involvement, control, and limit-setting—key features of the practice of authoritative parenting in adolescence that promotes positive developmental outcomes (Steinberg, 2001). Researchers agree that controlling behaviors in emerging adulthood are not appropriate and may interfere with emerging adults’ own goals and daily lives (Schiffrin & Liss, 2017), and scales that claim to measure helicopter parenting feature an assortment of behaviors appearing to capture control, overprotection, misguided support, intervening, and overinvolvement. Like most areas of psychology, helicopter parenting suffers from *proliferation* of constructs and measures (Anvari et al., 2025). This phenomenon occurs when accumulating instruments differ enough to blur lines between constructs and lead to difficulties in consolidating evidence within a field of study. For helicopter parenting or “overparenting,”^1^ there are now at least 16 stand-alone instruments (Zhang & Ji, 2023) but no consensus definition or organizing theoretical framework (Cui et al., 2022).

Padilla-Walker and Nelson (2012) define helicopter parenting as a type of control that features high warmth and support with low autonomy granting. Segrin and colleagues (2012) call it a “colloquial term” referring to excessive involvement and developmentally inappropriate behaviors that fail to promote autonomy. Luebbe and colleagues (2018) declare it to be a style of parenting that prevents autonomy development, overlapping with some existing constructs—behavioral and psychological control and monitoring, among others. Despite the lack of a common definition of helicopter parenting, all scale creators refer to one or more of the following criteria when describing helicopter parenting: (1) autonomy-limiting overinvolvement, (2) intervening to solve adult children’s problems for them, and (3) making decisions or otherwise doing things for the adult child that they should be capable of doing for themselves. In the absence of an existing consensus definition, we focus on these criteria as the core features of helicopter parenting for the purposes of the present study.

## Measure Proliferation and Item Diversity

Many items on existing helicopter parenting instruments align with the criteria described above (e.g., “my parent intervenes in solving problems with my employers or professors”). However, these are intermixed with topics such as instrumental support (e.g., “I help my child out with their transportation needs”), professional networking (e.g., “my parent looks for jobs for me”), emotional reactivity (“my parent overreacts when I encounter a negative experience”), and perceived parent feelings (“my parent feels like a bad parent when I make poor choices”). On one overparenting instrument, there is an entire subscale of items describing parents talking to their children about their interests and giving them advice (Segrin et al., 2012). In some cases this item diversity introduces conflicts between criteria for helicopter parenting: In two studies, subscales of items describing fixing problems, doing things for the adult child, and shielding them from adversity were correlated with autonomy-*supportive* parenting (Chevrier et al., 2023; Ryan et al., 2024). Without a consensus definition, nothing prevents helicopter parenting instruments from expanding to include all manner of parent involvement that occurs in emerging adulthood.

These observations call to mind the “jingle fallacy”, where numerous similarly-named scales are developed that capture distinct experiences, a problem even for mature constructs. Popular scales used to measure “depression”, for example, span 52 distinct symptoms, 21 of which appear on just one scale and on average, scales overlap in just one third of their item content (Fried, 2017). In other words, giving scales the same *title* does not mean that they measure the same *construct*—especially when item content is highly diverse. A consequence of the proliferation of similarly-named but diverse measures is that studies using measures of a presumed common construct find different associations with external variables. This leads to inconsistent findings within a literature and obstructs evidence synthesis (Anvari et al., 2025).

Measure proliferation and item diversity are important weaknesses that impede progress in the study of helicopter parenting. Most evidence of helicopter parenting’s ill effects is based on cross-sectional studies correlating scale scores with various psychological, social, and academic outcomes (see Cui et al., 2022 for a review). However, many studies find weak effects. In a meta-analysis of 52 studies testing associations with internalizing mental health symptoms, just 22% of associations calculated from the originating studies were significantly different from zero, indicating that most studies found no evidence of any association between helicopter parenting and internalizing symptoms (Zhang & Ji, 2023). Pooled correlations in that meta-analysis between helicopter parenting and depressive or anxious symptoms were around *r* = .15 (2% shared variance). These were also thought to be overestimates due to publication bias, suggesting the strength of the true associations might be even weaker. Some studies also show helicopter parenting to be associated with desirable academic outcomes (Luebbe et al., 2018; if paired with maternal warmth, Padilla-Walker et al., 2021). One recent study found that only the autonomy-limiting subscale of Luebbe and colleagues’ measure consistently predicted more emotion dysregulation, whereas information-seeking behaviors such as “my parent likes to have an update on my day-to-day life” predicted *less* emotion dysregulation (Nguyen et al., 2024). To the extent that weak and inconsistent findings in this literature are a consequence of excessive item content diversity, the problem began with incomplete scale validation efforts.

### Scale Validation

Establishing the construct validity of a measure demands attention to three types of evidence. The two familiar types are *structural validity*, whose evidence typically derives from strong inter-item correlations and factor analytic models, and *external validity,* whose evidence consists of strong associations of the measure with distinct constructs (Loevinger, 1957). The neglected third type of evidence is *substantive validity*, in which the goal is to establish a construct definition, outline its scope (including what it is *not*), and determine the content of items to be used for its measurement. Anecdotes about overly involved parents provided useful initial observations for a foundation on which to grow and clarify criteria for a helicopter parenting construct (conceptual enrichment; see Cronbach & Meehl, 1955), but articles introducing most helicopter parenting scales do not report taking any steps to formally validate item content. Instead, most studies that we reviewed skipped ahead to structural validity, basing their initial measure validation on the strength of factor loadings in orthogonal principal components analyses (LeMoyne & Buchanan, 2011; Schiffrin et al., 2014) or exploratory factor analyses (EFA; Padilla-Walker & Nelson, 2012; Bradley-Geist & Olson-Buchanan, 2014; Segrin et al., 2012).

One scale development study that implemented some substantive validity checks (Luebbe et al., 2018) began with a set of items from existing scales, asked PhD-level experts to generate additional items, and drew on undergraduates’ opinions of helicopter parenting through a focus group and text responses to a survey. However, they judged their scale to be externally valid in part because of high similarity to items appearing on existing measures, a problematic criterion if item content across existing scales is overly diverse. A later study (Schiffrin et al., 2019) found that scale scores from existing measures were intercorrelated, evidence in principle of external validity (Gonzalez et al., 2021). But these correlations were similar in strength to correlations of helicopter parenting with other, putatively distinct measures including psychological and behavioral control. Overall, validity evidence for helicopter parenting scales is mixed and concerns about measure proliferation are credible.

### Items Emphasizing Rare Behaviors

One consequence of the largely ad-hoc approach to scale development, influenced by anecdotes and media reports, is that many items on existing helicopter parenting scales suffer from floor effects, lowering power and compromising tests of associations with other measures. In one study that examined five pre-existing helicopter parenting scales (Schiffrin et al., 2019), mean scores were below the scale midpoint on every scale; some drastically so (e.g., 2.2 out of 7). Measures of helicopter parenting appear to disproportionately capture rarely-occurring behaviors, leading to a measure that is non-informative—or lacking precision—for most respondents (see Thissen & Orlando, 2001, p. 118 for a discussion of test information). Consequently, helicopter parenting correlations with other measures may be spurious and biased upward (Mader et al., 2023).

## Definitional Correspondence as a Means to Scrutinize Item Diversity

We contend that if the core features of autonomy limiting, intervening, and doing for the child are central to the definition of helicopter parenting, the construct cannot also be reliably and validly measured with instruments whose items collectively touch on virtually all aspects of the parent-child relationship in adulthood. At this still early stage of scholarly research on the topic, branches of study have not yet splintered into disconnected literatures. A logical next step is to seek evidence that will allow future scale refinement and consolidation around a narrowed set of items that exhibit *definitional correspondence*, defined as inter-rater agreement that an item corresponds to its construct’s definition (Colquitt et al., 2019). Most existing instruments use emerging adult self-report, and we focused on emerging adults as item judges in the present study for two additional reasons: (1) because parents and offspring disagree in their assessments of parenting behaviors (Korelitz & Garber, 2016; Wang & Hawk, 2023), and (2) because of concerns that parents present overly favorable impressions of their own parenting behaviors (Korelitz & Garber, 2016). Absent an existing consensus definition, in this study we created a lay-audience description of helicopter parenting that hinted at the focal criteria emphasized in other scale creators’ studies (autonomy-limiting, intervening, and doing for the adult child things they are capable of on their own). We then asked emerging adults to rate the correspondence of items drawn from existing scales with our description.

### Potential Sources of Definitional Non-Correspondence

Some emerging adults might rate an item as corresponding less well with a definition of helicopter parenting if they have prior experience with the behavior described in the item. Because exposure tends to be associated with more positive attitudes (e.g., Bornstein, 1989), emerging adults with prior item experience might be less inclined to ascribe the behavior to the construct, given the negative tenor of “helicopter parenting” references in the media. Indeed, educational test development work shows that item content familiarity influences how test-takers respond to items (Stricker & Emmerich, 1999).

Behaviors that constitute intrusive and autonomy-limiting parent involvement likely differ between younger, college-going emerging adults (the population on which every existing scale was developed) and their older and non-college counterparts. College graduates, for example, depend more on their parents for financial support in their early twenties, and transition to having their own children at a later age compared to their non-college peers (Mitchell & Syed, 2015). In one study, students in college perceived their parents as less intrusive than did their same-aged peers not in college (Nice & Joseph, 2023). Graduates in that study also viewed their parents as more supportive—and themselves as more self-reliant—than did their non-college peers.

## The Current Study

Focusing on helicopter parenting as perceived by emerging adults, the goal of this exploratory and descriptive study is to scrutinize items from existing measures and make recommendations for item inclusion in future measures. In a large sample of student and non-student emerging adults, we tested: (1) how much emerging adults agree that individual items describe helicopter parenting (definitional correspondence); (2) for which items correspondence levels differ between students and non-students; (3) for which items correspondence levels differ between emerging adults whose parents have and have not engaged in the behaviors described in the items; and (4) for which items correspondence ratings depend on the frequency with which the behaviors occur. Direct inquiries about item correspondence with orbiting constructs were beyond the scope of this study, but we did ask emerging adults to provide their impressions of parents’ motivations for engaging in the behaviors described in each item. Descriptions of helicopter parenting center on three reasons why parents remain involved with their adult children in developmentally inappropriate ways: to control their adult children; to provide support; or because parents worry their children will fail without intervention (Gao et al., 2025; Padilla-Walker & Nelson, 2012).

We anticipated that emerging adults would agree that items describing focal criteria referenced by previous scale creators—autonomy-limiting, intrusive behaviors and things emerging adults can do for themselves—qualify as helicopter parenting. Conversely, we anticipated that emerging adults would disagree that items describing necessary support, advice-giving, and non-intrusive involvement qualify as helicopter parenting. We also expected that students and emerging adults whose parents had previously engaged in behaviors described in the items would show lower agreement that those items described helicopter parenting. We expected that emerging adults’ feelings about whether parenting behaviors reflect control, support, or fear of failure would assist us in interpreting findings related to definitional correspondence, but we made no specific predictions. All data, code, and materials for this study are available online (https://osf.io/hc9qs).

## Method

### Participants

We recruited *n* = 1,464 participants between October and December, 2020, from a large Canadian university and from *Prolific.com*. Sample size and our decision to use a convenience sample of local undergraduates was dictated by time and resource limitations. Given the timing of data collection, most participants would have been subject to COVID-19 health and safety protocols (e.g., extended co-residence with parents). Between September and December 2020, participants completed a 20-min online questionnaire that included questions about helicopter parenting behaviors, and emerging adults’ opinions and experiences with these behaviors. University-recruited participants received course credit for their participation in the study. Participants from Prolific.com received $4. To ensure quality responses, we did not include participants in the analyses who: (1) completed 20% or more of the survey with a completion time under 360 seconds (three times the time needed to click through the survey without reading); (2) gave nonsense answers to an open-ended survey question; (3) did not complete any helicopter parenting item ratings, and (4) were under age 18 or were age 26 or older. This resulted in 1,222 participants retained for data analyses.

Table 1 provides a detailed breakdown of participants’ self-reported gender, race/ethnicity, and country of residence, presented separately for students and non-students. Most participants were students (70.2%), with an average age of 21.03 (SD = 2.35).

**Table 1.** Participants’ Self-Reported Gender, Race/Ethnicity, and Country of Residence

	Full sample *n*(%)	Students *n*(%)	Non-students *n*(%)
Total *n*	1,222	865	357
Gender *n*	1,221	864	357
Male	431 (35.3)	265 (30.7)	166 (46.5)
Female	766 (62.7)	590 (68.3)	176 (49.3)
Both	5 (0.41)	1 (0.11%)	4 (1.1%)
Neither	13 (1.1)	6 (0.69%)	7 (2.0%)
Other	6 (0.49)	2 (0.23%)	4 (1.1%)
Race/ethnicity *n*	1,180	826	354
White	675 (57.2)	479 (58.0)	196 (55.4)
Black	84 (7.1)	62 (7.5)	22 (6.20)
Brown	1 (0.08)	-	1 (0.28)
Asian	98 (8.3)	36 (4.4)	62 (17.5)
South Asian	53 (4.5)	49 (5.9)	4 (1.1)
West Asian	39 (3.3)	38 (4.6)	1 (0.28)
Southeast Asian	75 (6.4)	59 (7.1)	16 (4.5)
Indigenous	23 (1.9)	18 (2.2)	5 (1.4)
Latinx	27 (2.3)	15 (1.8)	12 (3.4)
Mixed	91 (7.7)	61 (7.4)	30 (8.5)
Other	14 (1.2)	9 (1.1)	5 (1.4)
Country of residence *n*	1,217	863	354
Canada	806 (66.2)	722 (83.2)	84 (23.7)
U.S.A.	376 (30.9)	106 (12.3)	270 (76.3)
Other	35 (2.9)	35 (4.1)	-

*Note.* Race/ethnicity categories appearing on surveys differed for Canada and U.S. residents based on country-specific census norms. Only U.S. residents saw “Brown” and “Asian” as options; for Canadian/Other, options were “South Asian”, “Southeast Asian”, and “West Asian”.

### Procedure

After completing demographic measures, we informed participants that they would see a series of statements “describing different behaviors that parents of young people sometimes engage in,” and that we were interested in their thoughts about each behavior. We then provided the following description:

“For the purposes of this study, our definition of helicopter parenting refers to parents of young people in their late teens and twenties, and it refers to things parents do that young people are capable of doing for themselves. We're thinking of parent behaviors that get in the way of young people living their lives and that might prevent them from becoming competent adults.”

Our team arrived at this description following back-and-forth discussions and revisions. Our goal was to write a general description for a lay audience that broadly referenced the criteria emphasized in other scale creators’ studies (autonomy-limiting, intervening, doing for the adult child) while avoiding direct references to actions described in the individual items participants would subsequently read. Participants saw this description paired with 10 randomly selected helicopter parenting item stems (described below). They were asked six follow-up questions pertaining to each stem.

### Measures

#### Helicopter Parenting Item Stems

We compiled a list of 54 different parenting behaviors that were used or trialed in six helicopter parenting scale development studies (i.e., LeMoyne & Buchanan, 2011; Luebbe et al., 2018; Odenweller et al., 2014; Padilla-Walker & Nelson, 2012; Schiffrin et al., 2014; Segrin et al., 2012).^2^ Original scales varied in structure from uni-to multi-dimensional, self- and parent-rated, and reflect both current and historical behaviors (i.e., while “growing up”), feelings, and attitudes. To avoid introducing construct-irrelevant method or wording variance (DiStefano & Motl, 2006; Gu et al., 2017), we edited original items to be structurally homogeneous. For example, the parent-rated item “I tell my child how to plan out certain activities” (Segrin et al., 2012) was edited to “My parent tells me how to plan out certain activities.” For the same reason, reverse-worded items were inverted if practical to eliminate negation, or excluded. We also excluded items that described feelings, thoughts about parents’ feelings, or that described parenting while growing up. We did not employ strict criteria in compiling the item list but prioritized items that described current behaviors. A complete list of item stems and their originating scales is available on our project page (https://osf.io/hc9qs).

#### Definitional Correspondence Ratings

Participants were asked six questions pertaining to each item. The first assessed definitional correspondence: “Based on the definition given above, how much do you think this statement describes helicopter parenting?” Response options were: 1 (*not at all*)*,* 2 (*a little*), 3 (*somewhat*), 4 (*moderately*), and 5 (*extremely*). The second question allowed for agreement to be conditional on behavior frequency: “How frequently would the behavior described in this statement have to take place for it to qualify as helicopter parenting?” Response options were: *not helicopter parenting regardless of frequency; only counts as helicopter parenting if it occurs very often,* and *definitely helicopter parenting regardless of frequency.*

#### Perceived Motivations for Parenting Behaviors

The next three questions asked for participants’ views about parents’ potential motivations for engaging in the behavior described in the item stem: *control* (“How much do you think the behavior described in this statement happens because a parent is trying to control their adult child’s life?”), *support* (“…because a parent is trying to be supportive or helpful toward their adult child?”), and *fear of failure* (“…because a parent is afraid their adult child will fail without their help?”). Response options for each question ranged from 1 (*not at all*) to 5 (*extremely*).

#### Personal Experience With Parenting Behaviors

Finally, we asked whether participants’ own parents had engaged in the behavior described in the item stem: “Is the behavior described in this statement something your own parents have done in the past year?” Response options were: *no*; *yes, my mother did this in the past year*; and *yes, my father did this in the past year*. Participants were considered to have prior experience with the described behavior if they reported it for either parent.

### Data Analysis

Each participant rated a randomly-selected subset of 10 item stems, giving sample sizes per item that ranged from *n* = 216 to *n* = 237. Random assignment of items to participants allowed us to limit respondent burden, assume a *missing completely at random* mechanism, and proceed with complete case analyses using available data. For each item, we computed a mean and 95% confidence interval based on participants’ responses to the question assessing definitional correspondence between the item and our provided definition of helicopter parenting. ANCOVAs for each item estimated definitional correspondence means and 95% confidence intervals separately for: (1) students and non-students, and (2) participants whose parents had or had not engaged in the behavior described in the rated item stem, adjusted for age. We used the Benjamini-Hochberg false discovery rate correction to account for familywise Type I error inflation within each family of 54 comparisons. Finally, we computed means and 95% confidence intervals of participants’ ratings for each perceived parent motivation.

## Results

### Definitional Correspondence

**Figure 1.**
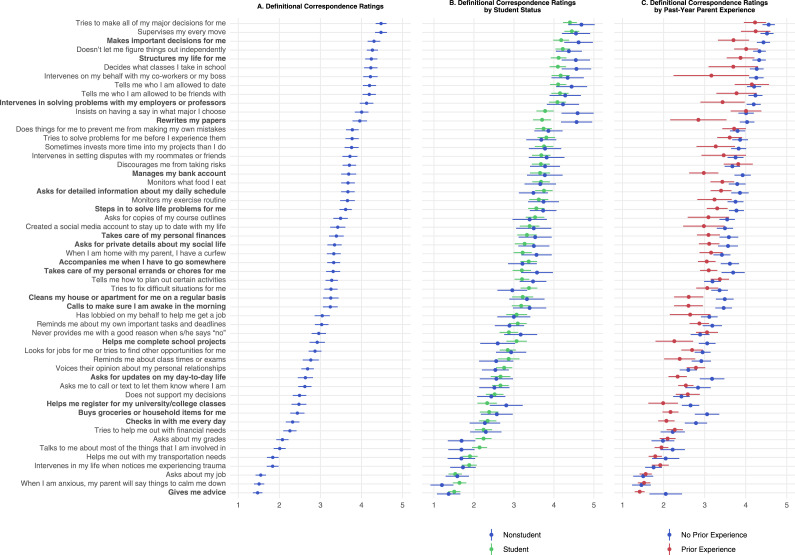
Mean definitional correspondence ratings and 95% confidence intervals for 54 helicopter parenting items, displayed in descending rank order from highest to lowest correspondence (Panel A). Definitional correspondence ratings are also displayed separately for students versus non-students (Panel B) and for emerging adults with versus without past-year parent experience of the behavior described in the item (Panel C; “prior experience” vs. “no prior experience”). Bolded items are those for which group differences in Panel C were statistically significant after correction for multiple tests (none of the differences in Panel B survived correction)

Figure 1, Panel A shows means and confidence intervals for participants’ definitional correspondence ratings. Items are ranked from highest to lowest mean correspondence, ranging from a high of 4.48 (between ‘moderately’ and ‘extremely’) to a low of 1.47 (rated between ‘not at all’ and ‘a little’). Items with high correspondence were primarily autonomy-limiting behaviors such as excessive supervision (e.g., “my parent supervises my every move”), enforced rules and structure (e.g., “my parent tells me who I am allowed to date”), and anticipatory problem-solving (e.g., “my parent does things for me to prevent me from making my own mistakes”). Items with low correspondence included advice and emotional support (e.g., “when I am anxious, my parent will say things to calm me down”), non-intrusive involvement (e.g., “my parent asks about my job”), and instrumental support for basic needs (e.g., “my parent buys groceries or household items for me”).

#### Student versus Non-Student Differences

Figure 1, Panel B shows estimated mean responses and confidence intervals of participants’ definitional correspondence ratings, separated by student and non-student status (adjusted for age). No items differed by student status after correction for multiple tests. Of the six items with nominal *p*-values below .05, four concerned academics.

#### Past Year Experience Present versus Absent

Figure 1, Panel C shows estimated mean responses and confidence intervals of participants’ definitional correspondence ratings, separated by whether participants’ parent(s) had engaged in the behavior described in the item stem in the past year. After correction for multiple tests, we observed significant mean differences between participants with and without past-year experience on 12 out of 54 items. On every item, participants with past-year experience of the behavior by their parent(s) agreed less strongly that the behavior represented helicopter parenting. Items for which the past-year experience difference was largest included several intervening and anticipatory problem-solving behaviors that were rare, for example: “My parent intervenes in solving problems with my employers or professors” (endorsed by 4.5% of participants who saw this item). Also among the largest differences were items describing common behaviors with much lower overall correspondence ratings, for example: “My parent buys groceries or household items for me” (reported by 69.5% of participants who saw this item).

#### Behavior Frequency

**Figure 2.**
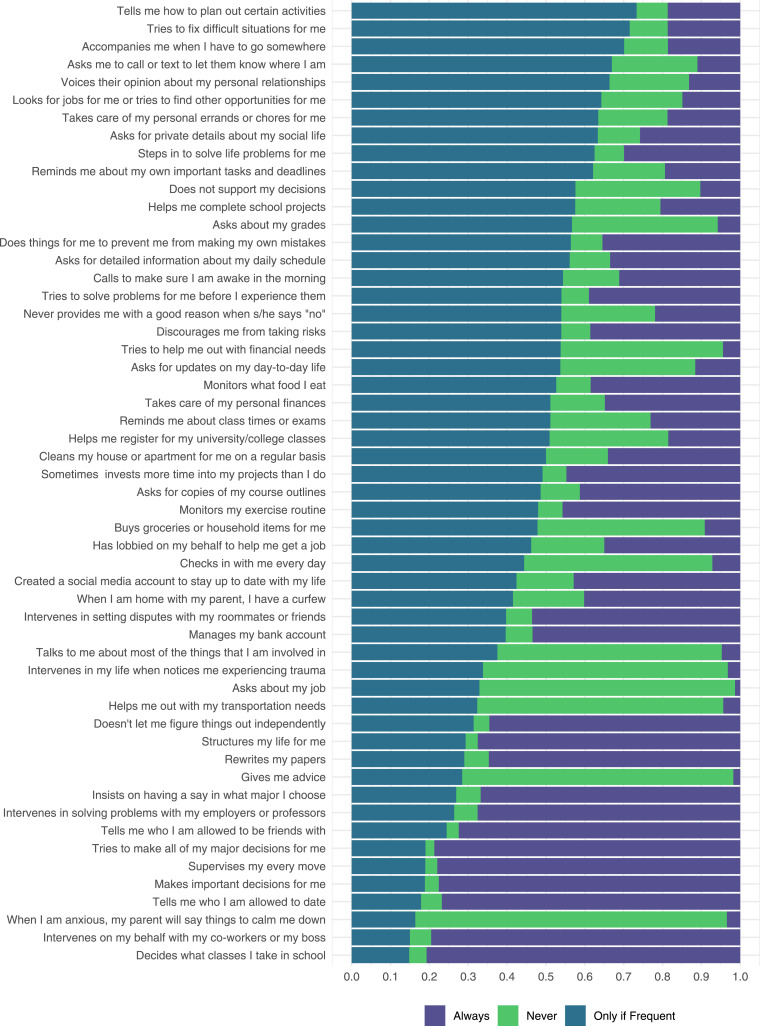
Ratings of 54 helicopter parenting items as corresponding to the description of helicopter parenting *always* (“definitely helicopter parenting regardless of frequency”), *never* (“not helicopter parenting regardless of frequency”), or *only if frequent* (“only counts as helicopter parenting if it occurs very often”). Items are displayed in descending rank order of endorsement of the “only if frequent” option

Participants reported that their parents engaged in the behavior described in a given item at rates ranging from 3.6% (“My parent intervenes on my behalf with my co-workers or boss”) to 91.6% (“My parent gives me advice”). Participants also varied in their opinions about whether behavior frequency mattered to item definitional correspondence. Figure 2 shows the proportions of participants who reported that each item was “always” or “never” helicopter parenting or that it qualified as helicopter parenting “only if frequent.” For 25 out of 54 items, more than half of participants felt that the behavior described in the item was helicopter parenting only if it occurred frequently (e.g., “My parent tells me how to plan out certain activities.”). For 14 items, more than half of participants felt that the behavior was *always* helicopter parenting, regardless of frequency (e.g., “My parent intervenes on my behalf with my co-workers or boss.”). For 6 items, more than half of participants felt that the behavior was never helicopter parenting (e.g., “My parent gives me advice.”). There were 9 items for which participants had more mixed opinions (i.e., no single response option was endorsed by at least 50% of participants), including items with large differences of opinion between participants with and without past-year experience, such as “My parent checks in with me every day”.

### Perceived Motivations for Helicopter Parenting

**Figure 3.**
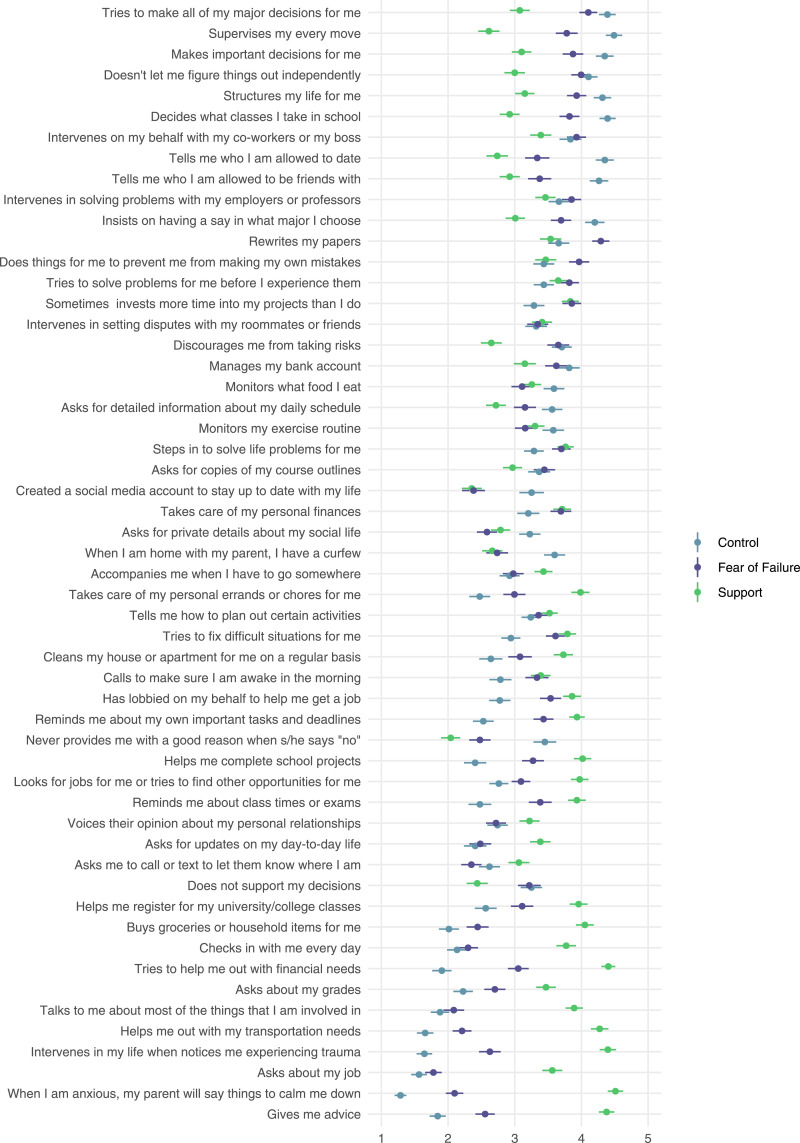
Mean perceived parent motivation ratings (control, fear of failure, and support) and 95% confidence intervals for 54 helicopter parenting items displayed in descending rank order from highest to lowest overall definitional correspondence. For each perceived parent motivation, items were rated on a scale ranging from 1 (strongly disagree) to 5 (strongly agree)

Figure 3 shows means and confidence intervals for participants’ responses to the questions assessing potential motives for parent behavior. In general, items with high definitional correspondence also had high agreement that parents’ motives were for *control* or *fear of failure*. Mean definitional correspondence ratings were correlated *r* = .94 with control motive ratings (*CI*_*95%*_ = .90, .97) and *r* = .82 with fear of failure motive ratings (*CI*_*95%*_ = .70, .89). The opposite was true for *support* motives; most items with high agreement that parents engage in the described behavior to be supportive or helpful had lower definitional correspondence (*r* = −.56, *CI*_*95%*_ = −.72, −.34). However, participants ascribed some level of support motive to nearly every item. For 45 out of 54 items, confidence intervals for support motive ratings included or surpassed the scale midpoint (“somewhat” or higher), and most remaining items were rated only slightly below the midpoint.

## Discussion

**Figure 4.**
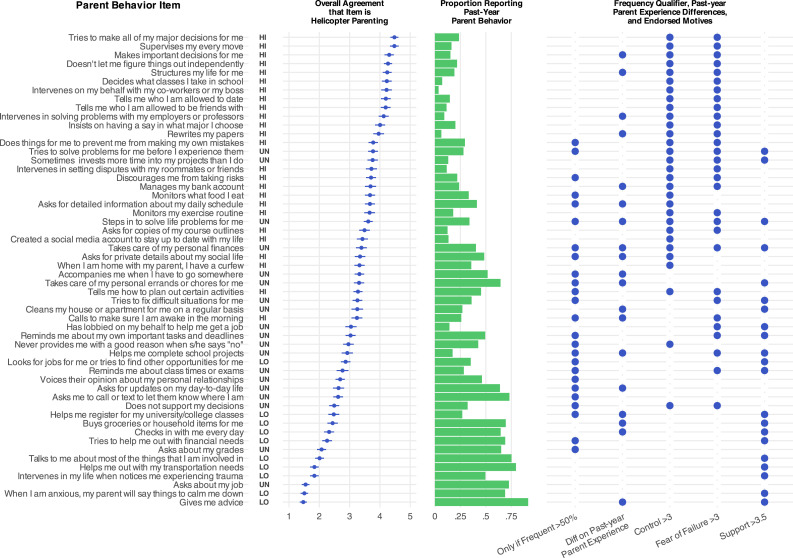
Global summary of results from the present study. Items are listed on the far left in descending rank order from highest to lowest overall definitional correspondence ratings, along with their classifications as either high correspondence (HI), low correspondence (LO), or unclassified (UN). The central bar chart shows proportions of participants reporting each item-described behavior by their own parent(s) in the past year. The final panel is a dot matrix summarising: which items were rated by >50% of participants as only meeting the definition of helicopter parenting if they occur frequently (frequency qualifier); which items had statistically significant differences between people with versus without past year parent experience of the behavior (past-year parent experience differences); which items had high control and fear of failure motive ratings with confidence intervals entirely above 3.0; and which items had high support motive ratings with confidence intervals entirely above 3.5 (endorsed motives). A dot indicates that the qualifier, difference, or motive was present for that item

This study provided a descriptive analysis of the definitional correspondence of a selection of 54 items used or trialed in one or more measures of helicopter parenting or overparenting. Figure 4 is a global summary of our work: Definitional correspondence ratings ranged from very low to very high, as did the proportion of people reporting that their parents actually engaged in the behaviors described in each item. However, ratings tended to be lower among emerging adults who directly experienced the behavior referenced in the item, compared to emerging adults with no direct experience. A further qualification was that emerging adults agreed that nearly half of items only corresponded to our description of helicopter parenting if the behaviors were frequently-occurring. Finally, items with higher definitional correspondence were often rated as motivated by attempts to control the adult child’s life or by fear the adult child will fail without help (and many items with *lower* correspondence were rated as motivated by intentions to be supportive or helpful). Pooling together all observations, we organize items into conceptual categories, describe key features of higher-versus lower-correspondence items, offer suggestions for future construct validation and scale development, and propose an interim construct definition.

### High Correspondence Items for Consideration in Future Scale Development

We filtered our item set down to those that had high definitional correspondence (items whose confidence intervals exceeded the scale midpoint of 3.0). Given the pattern of strong correlations between correspondence and motive ratings, we further narrowed the items to those that were also rated highly as arising from either control or fear of failure motives, and those that were not rated highly as arising from support motives (confidence intervals below 3.5 for the latter criterion). This resulted in 25 items that we organized into four categories: (1) *intrusive monitoring* (e.g., “My parent supervises my every move”); (2) *stepping in/intervening* (e.g., “My parent intervenes in settling disputes with my roommates or friends”); (3) *controlling decision-making* (e.g., “My parent structures my life for me”); and (4) *academic overinvolvement* (e.g., “My parent decides what classes I take in school”). Figure 4 flags each item classified as high correspondence (“HI”), and a complete list of our detailed classification scheme appears in our supplemental materials (https://osf.io/hc9qs). For 7 items, over half of participants felt that the behavior qualified as helicopter parenting only if it were frequently-occurring. For example, “My parent calls to make sure I am awake in the morning” may be intrusive if it’s a daily occurrence but welcome on special occasions when the consequences of sleeping in are severe (a major sports competition; an early morning flight).

High correspondence items appear on all six of the originating scales that we drew from, supporting prior observations of conceptual common ground across existing scales (Luebbe et al., 2018; Ryan et al., 2024). However, 8 out of 25 items had lower definitional correspondence ratings from emerging adults with past-year experience of the behavior described in the item, a potential source of measurement noninvariance that might undermine construct validity. Factors such as age, co-residence with parents, and cultural differences in family norms may contribute to determining which behaviors are reasonably universal exemplars of helicopter parenting and which depend on context. Participants in this study who were current students did not differ from non-students in their definitional correspondence ratings, perhaps because of demographic similarity between these groups or the possibility that many of the non-students were former students. In any case, student status does not appear to be a strong source of noninvariance.

### Low Correspondence Items to Consider Removing From Measures of Helicopter Parenting

Emerging adults in this study were also consistent in their views about which items did not fit well with the definition of helicopter parenting. We inverted our filtering scheme to instead limit items to only those with definitional correspondence confidence intervals *below* 3.0, *not* rated highly as arising from either control or fear of failure motives, and rated highly as arising from support motives (confidence intervals fully above 3.5). This resulted in ten items that we organized into three categories: (1) *non-intrusive monitoring* (e.g., “My parent talks to me about most of the things that I am involved in”); (2) *instrumental support* (e.g., “My parent helps me out with my transportation needs”); and (3) *mental health support* (e.g., “When I am anxious, my parent will say things to calm me down”).

All but one of the six originating scales we drew from included at least one low correspondence item, supporting concerns about measurement overdiversity and construct proliferation. This subset of low correspondence items generally did not describe controlling or autonomy-limiting parent behaviors, but did include problem-solving or intervening behaviors that some emerging adults may feel they are in fact *not* capable of doing for themselves. Providing tangible assistance to emerging adults is normative in North American families, implying a socially acknowledged need for this type of assistance (e.g., Fingerman et al., 2017). Buying plane tickets home for the holidays or filling the fridge when the adult child is between paychecks are examples of help parents provide. Given contemporary affordability challenges faced by emerging adults, this type of support is increasingly needed (Bluth & Manzoni, 2023). A recent exploratory study identified a factor consisting of items the authors described as “responsive overparenting” that shares commonalities with our low correspondence item set (Ryan et al., 2024). Given that this factor correlated positively with autonomy support, its items may not align with focal criteria for a helicopter or overparenting construct.

Similarly, some items appear to capture features of parent-child relationship quality rather than helicopter parenting, such as giving advice, talking about the adult child’s interests, and providing support when the adult child is feeling anxious. Versions of these items even appear on measures of relationship quality: the *Network of Relationships Inventory* (Furman & Buhrmester, 2009) includes items such as “How often do you depend on this person for help, advice, or sympathy?” (emotional support subscale). Valuing parents’ advice and involvement is clearly viewed by emerging adults as positive, and even intense support (occurring several times per week) was associated in one study with greater adjustment and life satisfaction (Fingerman et al., 2012).

### Unclassified Items

The filtering schemes we used to summarize high- and low-correspondence items left 19 items (35.2%) unclassified. Five of these had low definitional correspondence, but unlike the other low-correspondence items summarized above, they had *low* ratings on perceived support motivations (e.g., My parent asks for updates on my day-to-day life”). Five others had high definitional correspondence, but unlike the other high-correspondence items summarized above, they had *high* ratings on perceived support motivations (e.g., My parent tries to solve problems for me before I experience them”). Six other items also had high support ratings and weren’t classifiable for at least one other reason, exposing a pattern raised in other research that helicopter parenting comingles control and support (Padilla-Walker & Nelson, 2012). Only 9 out of 54 items had confidence intervals for support motives below 3.0 (scale midpoint), and every item had a mean support rating above 2 out of 5. It may be that most emerging adults assume that parents are trying help in whatever they do, even if their actions are unwelcome.

Some unclassified items may be useful complements to the prototypical but rare exemplars of helicopter parenting. For example, “My parent takes care of my personal errands or chores for me” had a modest definitional correspondence rating (3.31, *CI*_*95%*_ = 3.14, 3.48), participants did not strongly rate this behavior as motivated by control or fear of failure but did see it as supportive (3.99, *CI*_*95%*_ = 3.85, 4.12), and 64.4% of participants’ parents did this in the past year.

### Frequency Considerations for Items Included in Helicopter Parenting Instruments

Nearly half of items in this study were judged by at least half of participants to qualify as helicopter parenting “only if it occurs very often.” The concept of helicopter parenting arose from the notion that some parents might be too involved in their adult children’s lives. Yet this notion doesn’t clarify when and how parents cross the line from appropriately-to over-involved. Is a parent who “asks about my grades” once after midterms and again toward the end of semester engaging in helicopter parenting, or just interested in their child’s academic success? Are parents hovering if they “ask me to call or text to let them know where I am”, even if it’s only because the child is heading off on a road trip to a part of the country they’ve never visited? Participants in the present study clearly see the distinction between occasional and regular involvement, and these results call into question the value of including items on a measure of helicopter parenting that could be interpreted differently depending on the regularity of the behaviors. The same concerns apply to items describing one-time events such as “choosing a major” and “creating a social media account” that may not suit a set of items attempting to measure an ongoing pattern of behavior. Future work refining measures of helicopter parenting may wish to incorporate frequency information directly into item stems or consider tailoring scale response options to capture frequency.

Figure 4 also shows that many of the items with the strongest definitional correspondence were behaviors that few parents actually engaged in. Some were so rarely reported that their presence on a composite scale might be more harmful than helpful. To illustrate, suppose we wished to evaluate the reading proficiency of a group of first-grade children but used only texts rated for eighth-grade children to do so. Our resulting scores would incorrectly (and uninformatively) tell us that nearly all children are very low in reading proficiency. Ideally, a psychometric scale comprises items that capture—with adequate precision—the full range of experiences needed to define the construct (Embretson & Reise, 2000, pp. 183–185). By overemphasizing items that describe alarming but rare behaviors, helicopter parenting scales lose predictive value.

### Summary

Based on our findings, we offer an interim definition of helicopter parenting that summarizes our observations from the present study. Our definition is limited in part by the criteria we focused on in the present study and the items that participants rated. We present it in the interest of providing a useful starting point for item development and selection in future scale refinement, for lines of research on both helicopter parenting and overparenting:

Parents of emerging adults may be described as engaging in helicopter parenting if they act toward their children in a way that exerts developmentally inappropriate control, limits their autonomy, unnecessarily resolves problems on their behalf, or intervenes in their private affairs. Parents’ actions may be unwelcome but recognized as motivated by a desire to provide support. Helicopter parenting is not involvement in day-to-day functioning that is better described as providing emotional support or engaging in a mutually reciprocated relationship with the adult child. Some involvement may be experienced as helicopter parenting if it occurs too frequently, whereas some actions that appear to control, limit autonomy, problem-solve, or intervene may not reflect helicopter parenting if they occur rarely or are welcome and appropriate given the context for the action.

For researchers interested in learning more about conducting scale development and refinement, a list of resources on best practices is available in Fried and Flake (2020).

### Limitations

Our descriptive approach to this study is limited by a lack of prespecified criteria for decisions we made during the design and analysis phases. Our list of items and the description of helicopter parenting we provided to participants were the result of team conversations and judgement calls. Our lay description of helicopter parenting hinted at autonomy-limiting and intervening actions that are criteria referenced by existing informal definitions, and may have primed our participants to think of helicopter parenting too narrowly if indeed it is a much broader construct (e.g., with “demanding” and “responsive” expressions; Ryan et al., 2024). Analyses were also exploratory, and we made decisions about how to organize and report our findings after already examining the data. To mitigate concerns about rigor, the complete data and analysis code for this study are publicly available. In line with the approach taken by Carpenter (2016), an appropriate item content validation step not taken in the present study would be to ask participants to match items with different, orbiting construct definitions (see also Anderson & Gerbing, 1991).

An issue that was beyond the scope of the present study was mother versus father differences in how helicopter parenting is measured. Some studies have asked participants about their mothers’ and fathers’ behaviors separately, producing evidence that measures may not be invariant across parents (Crapo et al., 2021). Focusing on emerging adults’ perspectives, our view is that we first need to resolve whether or not helicopter parenting is a global construct producing similar lived experience for the impacted young person, regardless of which parent(s) engage in the behaviors described by scale items. Yet, parents and their offspring generally disagree when assessing parenting (Hou et al., 2020), so it remains unclear whether helicopter parenting is present if parents report behaviors stemming from their own preoccupations with their near-adult children even if from the child’s perspective the parent isn’t acting intrusively or limiting their sense of autonomy. A psychometrically strong self-report measure will not on its own be sufficient to fully understand helicopter parenting. Multiple-informant (parent-child) measurement studies are a valuable next step after resolving validity concerns.

This study also considered only two potential sources of variation in definitional correspondence (student vs. non-students; emerging adults with vs. without prior item experience) and adjusted for age. From a measurement perspective, it is good news that students and non-students rated items similarly. A core assumption made when using a multi-item scale to compare people on a measured construct is that the items bear the same relationship to the construct regardless of external differences (Meredith, 1993). To support the valid use of a measure, at least some items must be non-invariant across external factors such as age, gender, and other characteristics that influence item responding (Reise et al., 1993). In the present study, safety protocols related to the COVID-19 pandemic (including extended co-residence with parents) are one unique source of influence. Items that we found to differ between students with and without prior item experience may compromise the reliability and validity of the measures they contribute to. Future research should examine other sources of plausible item-level invariance, such as whether a young person lives at home with their parents and whether cultural differences dictate what counts as normative parent involvement.

Culture is one likely source of non-invariance that is conspicuously absent from the present study, and research shows that emerging adults in collectivistic cultures (e.g., China, Korea) experience helicopter parenting as less harmful or threatening compared to their counterparts from the largely individualistic Western world (Hong & Cui, 2023; Lee & Kang, 2018). Cultural norms undoubtedly dictate what parent behaviors are considered acceptable, and these norms may have consequences for measurement invariance as described above. In the present study we assessed only participants’ ethnic backgrounds and are unable to test for cultural differences in definitional correspondence.

### Conclusion

Across a corpus of 54 items, we showed that emerging adults had widely differing views of items’ correspondence with a provided description of helicopter parenting. In a literature saturated with helicopter parenting scales and little critique of measurement quality, this study addressed an urgent need to scrutinize existing items. How helicopter parenting fits into the network of existing dimensions of parenting, however, remains an open question. Is helicopter parenting a multidimensional or unidimensional construct, and is it distinct from similarly-titled constructs (overparenting, indulgent parenting)? Our findings of high overlap between participants’ ratings of definitional correspondence and their endorsement of control and fear of failure motives raise the possibility that helicopter parenting might be a special case of existing concepts such as behavioral control and monitoring (Padilla-Walker & Nelson, 2012).

In our enthusiasm for understanding this emergent construct, researchers must take care to avoid creating and using measures that consolidate all facets of parenting during the transition to adulthood under the banner of helicopter parenting or overparenting. Doing so fails to distinguish the developmentally protective and promotive aspects of ongoing parent-child relationships from truly intrusive and autonomy-limiting practices. Development of a new measure, or refinement of an existing measure of helicopter parenting may benefit from results of this study suggesting low correspondence items to rule out, factoring in behavior frequency when designing items, and excluding items that are better indicators of other facets of the parent-child relationship.

In an ironic turn of events, one journalist who reported on the scourge of helicopter parenting (Strauss, 2006) walked back her comments nearly 10 years later—shortly after the scales reviewed in the present study were first published—noting that instances of highly intrusive parenting in emerging adulthood are too rare to treat as a pervasive problem (Strauss, 2015). Rarity was a recurring theme in the present study for many stereotyped behaviors, and we agree with Brown and colleagues (2024) that responsive, involved parenting in moderation will set young people up for success.

## Supplemental Material

Supplemental Material - Construct Diversity in Measures of Helicopter Parenting in Emerging Adulthood Supplemental Material for Construct Diversity in Measures of Helicopter Parenting in Emerging Adulthood by Andrea. L. Howard, Sydney Bota, and Megan Lamb in Emerging Adulthood

## Notes

1. The terms *helicopter parenting* and *overparenting* have been used interchangeably and both lack a consistent definition. We use the term helicopter parenting throughout this paper and understand the two terms to be compatible and capture similar ideas.

2. These six scales were the ones our team was able to find in mid-2020 via literature search and that were developed to measure helicopter parenting in emerging adults.
